# Knockout of TSC2 in Nav1.8+ neurons predisposes to the onset of normal weight obesity

**DOI:** 10.1016/j.molmet.2022.101664

**Published:** 2022-12-28

**Authors:** Jennifer M. Brazill, David Shin, Kristann Magee, Anurag Majumdar, Ivana R. Shen, Valeria Cavalli, Erica L. Scheller

**Affiliations:** 1 Department of Medicine, Division of Bone and Mineral Diseases, Washington University, Saint Louis, MO, USA; 2 Department of Neuroscience, Washington University, Saint Louis, MO, USA; 3 Center of Regenerative Medicine, Washington University School of Medicine, Saint Louis, MO, USA; 4 Hope Center for Neurological Disorders, Washington University School of Medicine, Saint Louis, MO, USA; 5 Department of Cell Biology and Physiology, Washington University, Saint Louis, MO, USA; 6 Department of Biomedical Engineering, Washington University, Saint Louis, MO, USA

**Keywords:** mTOR, Bone, High fat diet, Skinny fat, Normal weight obesity, Sensory nerve, Nav1.8, Neurometabolism, Anxiety, Itch

## Abstract

**Objective:**

Obesity and nutrient oversupply increase mammalian target of rapamycin (mTOR) signaling in multiple cell types and organs, contributing to the onset of insulin resistance and complications of metabolic disease. However, it remains unclear when and where mTOR activation mediates these effects, limiting options for therapeutic intervention. The objective of this study was to isolate the role of constitutive mTOR activation in Nav1.8-expressing peripheral neurons in the onset of diet-induced obesity, bone loss, and metabolic disease.

**Methods:**

In humans, loss of function mutations in tuberous sclerosis complex 2 (TSC2) lead to maximal constitutive activation of mTOR. To mirror this in mice, we bred Nav1.8-Cre with TSC2^fl/fl^ animals to conditionally delete TSC2 in Nav1.8-expressing neurons. Male and female mice were studied from 4- to 34-weeks of age and a subset of animals were fed a high-fat diet (HFD) for 24-weeks. Assays of metabolism, body composition, bone morphology, and behavior were performed.

**Results:**

By lineage tracing, Nav1.8-Cre targeted peripheral sensory neurons, a subpopulation of postganglionic sympathetics, and several regions of the brain. Conditional knockout of TSC2 in Nav1.8-expressing neurons (Nav1.8-TSC2^KO^) selectively upregulated neuronal mTORC1 signaling. Male, but not female, Nav1.8-TSC2^KO^ mice had a 4–10% decrease in body size at baseline. When challenged with HFD, both male and female Nav1.8-TSC2^KO^ mice resisted diet-induced gains in body mass. However, this did not protect against HFD-induced metabolic dysfunction and bone loss. In addition, despite not gaining weight, Nav1.8-TSC2^KO^ mice fed HFD still developed high body fat, a unique phenotype previously referred to as ‘normal weight obesity’. Nav1.8-TSC2^KO^ mice also had signs of chronic itch, mild increases in anxiety-like behavior, and sex-specific alterations in HFD-induced fat distribution that led to enhanced visceral obesity in males and preferential deposition of subcutaneous fat in females.

**Conclusions:**

Knockout of TSC2 in Nav1.8+ neurons increases itch- and anxiety-like behaviors and substantially modifies fat storage and metabolic responses to HFD. Though this prevents HFD-induced weight gain, it masks depot-specific fat expansion and persistent detrimental effects on metabolic health and peripheral organs such as bone, mimicking the ‘normal weight obesity’ phenotype that is of growing concern. This supports a mechanism by which increased neuronal mTOR signaling can predispose to altered adipose tissue distribution, adipose tissue expansion, impaired peripheral metabolism, and detrimental changes to skeletal health with HFD – despite resistance to weight gain.

## Highlights

• Nav1.8-TSC2^KO^ mice are a model of mTOR activation in Nav1.8-lineage neurons.
• Nav1.8-TSC2^KO^ mice develop normal weight obesity with bone loss and altered fat distribution when fed high-fat diet.
• Nav1.8-TSC2^KO^ mice also have signs of chronic itch and anxiety-like behavior.
• Excess neuronal mTOR signaling may drive diet-induced fat expansion, poor metabolism, and bone loss – despite resistance to weight gain.

## Graphical abstract

**Figure:**
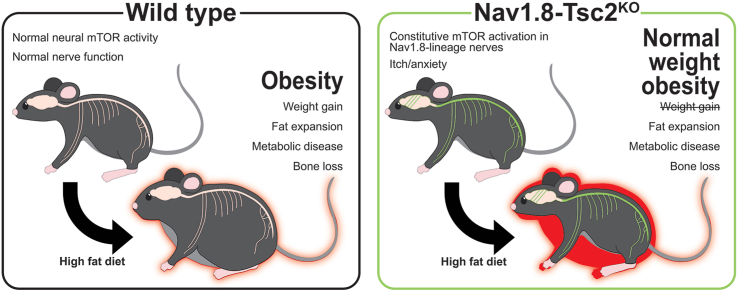

## 1 Introduction

Tuberous sclerosis complex 2 (TSC2) is a tumor suppressor that forms the core tuberous sclerosis complex with TSC1 [1,2]. The TSC1/TSC2 complex integrates nutritional, growth factor, and hormonal signals to regulate the biologic activity of the mammalian target of rapamycin (mTOR). Overall, this controls diverse aspects of cell growth and function through phosphorylation-mediated activation of downstream effectors by the two unique mTOR-containing protein complexes, mTORC1 and mTORC2 [[1], [2], [3]]. Loss of function mutations in TSC1 and/or TSC2 lead to maximal constitutive activation of mTOR, particularly mTORC1. In humans, this causes an autosomal dominant condition referred to as ‘tuberous sclerosis complex’ that is associated with the development of tumors and a range of neurological and psychiatric symptoms, including seizures and a high prevalence of autism spectrum disorders (7). In mice, destabilization of the TSC1/TSC2 inhibitory complex through conditional knockout of TSC1 or TSC2 is a well-established model to study the cell- and tissue-specific consequences of constitutive mTORC1 activation [[4], [5], [6], [7]].

Clinical inhibitors of mTORC1, including sirolimus, everolimus, and temsirolimus, have been in use since 1999 and represent three of the 52 FDA-approved small molecule protein kinase inhibitors [8]. Approved clinical uses include immunosuppression to prevent rejection of transplanted organs and treatment of certain cancers [8]. Beyond this, the list of conditions that may benefit from modulation of mTOR-dependent pathways is continually expanding due to the tight control of cellular metabolism, growth, and function by mTOR-containing complexes. This includes efforts to translate mTOR regulation as a therapeutic for neurological disorders, metabolic disease, and skeletal health [[9], [10], [11], [12]]. Metformin, for example, is a common anti-diabetic therapy that has mTOR inhibitory properties among other functions [13,14]. A key hurdle limiting this clinical translation is our incomplete understanding of the functional consequences of cell type-specific mTOR activation on these outcomes. Prior work in this area has primarily focused on the functions of mTOR in pancreatic β-cells, visceral organs, adipocytes, and within the brain [[15], [16], [17], [18], [19], [20]]. Our project extends these studies by considering the role of constitutive mTOR activation through conditional TSC2 knockout in Nav1.8+ neurons.

Nav1.8 is a voltage-gated sodium channel that is highly expressed by peripheral sensory neurons [[21], [22], [23], [24]]. This includes sensory nerve populations in the nodose (82%+), dorsal root (80%+), and trigeminal ganglia (67%+) [21]. We selected the Nav1.8-Cre model for our study based on its relative specificity for the sensory nervous system and its capacity to modify the mTOR-mediated interoceptive pathways controlling the metabolic responses to diet. Prior studies on the function of mTOR in the peripheral nervous system have centered on neuronal development and axon regeneration. This includes three prior models of TSC1 or TSC2 knockout. In the first, Synapsin-Cre was used to generate a pan-neuronal knockout of TSC1. These mice experienced delayed development, seizures, and a reduced median survival time of 35 days, likely due to targeting of the brain [7]. By contrast, TSC2 knockout with either Advillin-Cre or Nav1.8-Cre did not impact survival and improved axon regeneration after injury [[4], [5], [6]]. Beyond this, the capacity for mTOR activation in the peripheral nervous system to influence metabolic and skeletal health remains unexplored.

There is a well-established observation that obesity and nutrient oversupply increase mTOR signaling in multiple cell types and organs [2,10,16,25]. This may contribute to the onset of insulin resistance and other consequences of chronic metabolic disease [25]. By contrast, caloric restriction and endocrine longevity factors often decrease mTOR [26]. Despite these observations, it remains unclear when and where mTOR activation mediates these effects. We hypothesized that mTOR signaling in the Nav1.8-lineage neurons may represent a key point of metabolic control. To test this hypothesis, we bred Nav1.8-Cre mice with TSC2^fl/fl^ animals to conditionally deplete TSC2 in Nav1.8-expressing neurons. Male and female mice were studied from 4- to 34-weeks of age and a subset of animals were fed a high-fat diet (HFD) for 24-weeks. We then performed assays to measure metabolism, body composition, bone morphology, and behavior.

## 2 Material and methods

### 2.1 Mice

Experiments were approved by the Animal Studies Committee at Washington University. TSC2^fl/fl^ (floxed allele; MGI:3712786 [27]), Nav1.8^Cre/+^ (MGI:3042874 [24]), Rosa26-ZsGreen (Ai6, JAX:007906 [28]), and Rosa26-tdTomato (Ai9, JAX:007909 [28]) mice were as described previously. To generate mice with TSC2 knockout in Nav1.8 expressing neurons, Nav1.8^Cre/+^; TSC2^fl/fl^ males were crossed with Cre negative TSC2^fl/fl^ females. Male and female control (Cre negative; TSC2^fl/fl^) and conditional knockout (Nav1.8^Cre/+^; TSC2^fl/fl^) littermates were used for all assessments. To generate Ai6 (ZsGreen, green fluorescent protein - GFP) or Ai9 (tdTomato) lineage reporter animals, Nav1.8^Cre/+^ males were bred with homozygous Ai6 or Ai9 reporter mice. Mice were group housed in a specific pathogen-free facility at 22–23 °C on a 12-hour light/dark cycle and fed standard chow (LabDiet® PicoLab®, 5053; 4.07 kcal/g) or 60% high-fat diet (HFD, Research Diets, D12492; 5.21 kcal/g) *ad libitum*. HFD was administered for 24-weeks, from 10- to 34-weeks of age.

### 2.2 Management of itch-related lesions

As part of our initial monitoring, we observed that a subset of Nav1.8-TSC2^KO^ mice developed itch-related skin lesions near the ears and base of the neck. Rear nail trimming was performed when lesions were observed to limit further damage, and lesions were treated with topical triple antibiotic ointment to promote wound healing. Occasionally, the sudden onset and severity of the lesions was sufficient to warrant euthanasia. To reduce the incidence of itch-related lesions for our longer-term HFD studies, we employed weekly nail trimming and monitoring for the Nav1.8-TSC2^KO^ mice. This was designed to limit any lesion-associated morbidity in the animals without modifying underlying changes in nerve function or the neuropsychological condition.

### 2.3 Lineage tracing of Nav1.8-Cre targeted cells

Nav1.8^Cre/+^; Ai9^+/−^ and Nav1.8^Cre/+^; Ai6^+/−^ mice were used for lineage tracing of Nav1.8-Cre targeted cells using previously described methods [29]. Briefly, mice were deeply anesthetized with ketamine/xylazine prior to perfusion with 10 mL of PBS followed by 10 mL of 10% neutral buffered formalin through the left ventricle of the heart. Perfused tissues were post-fixed for 24-hours in 10% neutral buffered formalin prior to washing and infiltration with 30% sucrose. Whole tissues, harvested from mice with Ai6/GFP fluorescent signal including tibia, adrenal gland, kidney, liver, pancreas, spleen, and adipose tissue, were embedded in optimal cutting temperature (OCT) compound for cryosectioning at 50 μm thickness (Tissue-Tek, 4583). Sections were immunostained with anti-GFP antibody (Abcam, ab13970; 1:1000; secondary Jackson ImmunoResearch, 703-545-155, 1:500) to amplify endogenous signals, and with DAPI to visualize nuclei (Sigma Aldrich, D9542). Tiled serial images of stained sections were taken with a 10× objective using a Nikon spinning disk confocal microscope (μm/px = 0.650, step size = 5 μm, number of steps = 10, overlap = 10%) and analyzed in ImageJ/FIJI [30]. Step-by-step tissue preparation and immunostaining instructions are also available at protocols. io (DOI: dx.doi.org/10.17504/protocols.io.bqu2mwye).

Brains and spines were also isolated from perfused Ai9/TdTomato mice. Whole thoracic spines were decalcified for 2-weeks in 14% ethylenediaminetetraacetic acid (EDTA) at pH 7.2. Spines were then embedded in OCT and cut transversely at 50 μm thickness to simultaneously visualize the spinal cord, dorsal root ganglia (DRG), paravertebral ganglia, and surrounding bone/tissues. Spine sections were immunostained with anti-tyrosine hydroxylase (TH, Abcam ab152; 1:1000; secondary Jackson ImmunoResearch, 711-545-152, 1:500), anti-calcitonin gene related peptide (CGRP, Bio-rad, 1720-9007; 1:1000; secondary Jackson ImmunoResearch, 705-605-147, 1:500), and DAPI as described previously [29]. Sections were imaged as detailed above. The endogenous Ai9/TdTomato signal did not require amplification. Whole brains were frozen at −80 °C prior to cutting into 6-series of 50 μm free-floating sections using a Microm HM 400 microtome on a Physitemp controller at −40 °C. Sections were stained with DAPI and mounted onto glass slides. Nav1.8^Cre/+^; Ai9^+/−^ brain sections were imaged using both a Nanozoomer scanner for the initial survey of whole-slide results and a Nikon Spinning Disk confocal microscope (10×, μm/px = 0.650, step size = 5 μm, number of steps = 3, overlap = 10%) to obtain tiled, high-resolution images of select representative sections. Brain regions were identified using the Allen Brain Atlas mouse p56 Sagittal and Coronal reference atlases [31].

### 2.4 Western blot

For Western blot analysis of protein expression, following euthanasia with carbon dioxide and cervical dislocation, tissues were homogenized by bullet blender with 0.5 mm zirconium oxide beads in SDS protein lysis buffer (1% SDS, 60 mM Tris-HCl, pH 8.0, 12.5 mM EDTA) with 1× protease inhibitor (Sigma) and phosphatase inhibitors (1 mM PMSF, 1 mM Na_3_VO_4_, and 1 mM NaF). Lysates were boiled at 95° for 5 min and then centrifuged at 13,000×*g* for 20 min at 4° prior to transfer of supernatants to a clean tube. Protein concentration was measured by Pierce BCA assay (Thermo Scientific) and equalized with lysis buffer to 0.3 μg/μL for DRG and 1 μg/μL for bone. Samples were denatured and reduced with 4× NuPAGE LDS Sample Buffer with 2.5% β-mercaptoethanol by heat at 70° for 10 min. Equal protein was loaded per tissue type (8 μg DRG and 30 μg bone) alongside EZ-Run Pre-Stained Rec Protein Ladder (Fisher) into NuPAGE 4–12% BisTris Midi Gels, 1.0 mm x 20 wells (Invitrogen). SDS-PAGE was performed using a Midi Gel Adapter (Invitrogen) in a Criterion Cell and XCell II Blot Module (Bio-Rad) per NuPAGE user guide instructions. Proteins were transferred to a PVDF membrane using the XCell II Blot Module (Bio-Rad). Membranes were blocked in 5% milk in Tris buffered saline + 0.5% TWEEN (TBST) for 1 h at RT with gentle agitation, washed 3× with TBST, and incubated with primary rabbit IgG antibodies diluted in 5% BSA in TBST overnight at 4° with gentle agitation. Primary antibodies and dilutions follow: TSC2 (Cell Signaling 4308; 1:1,000), S6 ribosomal protein (Cell Signaling 2217; 1:1,000), phospho-S6 (Ser240/244; Cell Signaling 5364; 1:750), α-tubulin (ProteinTech 11224-1-AP; 1:5,000). Membranes were then washed 3 × 5 min in TBST before incubation with Alexa 680-conjugated anti-rabbit secondary antibody (Jackson IR 11-625-144; 1:10,000) diluted in 5% BSA in TBST for 1 h at RT with gentle agitation. Following 3 × 5 min washes in TBST, proteins were detected with the Odyssey Infrared Imaging System (LI-COR Biosciences).

### 2.5 Body size, body composition, food intake, and tissue mass

Body mass was assessed on a weekly basis starting at 4-weeks of age using a digital scale. Body composition was measured using whole-body quantitative magnetic resonance with an EchoMRI-900 (Echo Medical Systems). Briefly, animals were placed into a thin-walled plastic cylinder with an insert added to limit movement prior to scanning with a low-intensity (0.05 T) electromagnetic field to measure fat, lean mass, free water, and total body water. Food intake was assessed weekly by weighing the food at the start of the week and then again at the end of the week. Weekly intake was normalized to the number of mice in the cage per day. Mice were housed based on genotype to facilitate this analysis. Any food pieces on the floor were recovered and included in this measurement. In the case of excessive grinding or shredding of food, the measurement was excluded from the analysis. At the endpoint dissection, body and tibia length were measured, and organs including fat, liver, spleen, and testicles or uterus were dissected and weighed.

### 2.6 Glucose and insulin tolerance testing

Mice were fasted on aspen bedding for 6 h. For glucose tolerance testing (GTT), 25% dextrose in saline was administered at 2 g/kg by intraperitoneal injection (IP). For insulin tolerance testing (ITT), 0.1 U/mL of insulin was administered IP at 0.75 U/kg. Blood glucose was measured from the tail-vein with a glucometer (Bayer Contour) at baseline and at the indicated times following injection of glucose (GTT) or insulin (ITT). GTT and ITT tests were performed at least 4 days apart.

### 2.7 Bone morphology

Mice were anesthetized with 1–2% isoflurane and placed into the scanning bed of a VivaCT40 (Scanco Medical) for micro-computed tomography. The right limb was straightened and stabilized with the foot secured to optimize positioning for the tibia. Scans were taken at 70 kVp; 114 uA; 8 W within a 21.5 mm field of view at 21 μm voxel resolution. The cortical bone scan region was set 1 mm proximal to the tibiofibular junction and trabecular scan region was set distal to the metaphyseal growth plate. Each scan region spanned a ∼2 mm segment so that the scan time was limited to 6.7 min per region. Analysis was performed using Scanco software and was restricted to 20 slices (420 μm) of the cortical bone by morphology and 50 slices (1 mm) of the trabecular bone in the metaphysis immediately distal to the growth plate.

### 2.8 Measurement of plasma corticosterone

Mice were fasted on aspen bedding for 6 h. Whole blood was collected retro-orbitally with heparin-coated glass capillary tubes from mice anesthetized with 1–2% isoflurane between ZT7 and ZT10. Blood was immediately extruded into EDTA-coated sample tubes, incubated on ice for 20 min, then centrifuged at 3,000×*g* for 15 min at 4 °C. Plasma supernatant was then stored at −80 °C until use. Plasma corticosterone was measured with the Enzo Corticosterone ELISA kit (Enzo Life Sciences, ADI-900-097) per the manufacturer's instructions.

### 2.9 Behavioral assessments

Behavioral studies were completed in mice that had been raised on control chow diet since weaning. To quantify the onset of itch-related skin lesions, control chow-fed mice were tracked from weaning at 3- to 4-weeks of age until 12-weeks of age. Locomotor and anxiety-related behavior were assessed by open-field testing. Eight-week-old male and female mice were evaluated over a 1-h period (6 × 10 min blocks) in a Plexiglass open-field arena (41 × 41 × 38.5 cm high) equipped with computerized photobeam instrumentation (Kinder Scientific, Poway, CA) as previously described [32]. MotorMonitor software (Kinder Scientific, Poway, CA) was used to record and analyze beam-break data. Locomotion was analyzed by total ambulations (whole-body movements), vertical rearing frequency, and distance traveled. Anxiety-related behavior was evaluated by dividing the open field into a central zone (25.6 × 25.6 cm) and a surrounding peripheral zone (7.7 cm wide) and analyzing time spent, distance traveled, and number of entries into the periphery versus the center zone. Thigmotaxis, the tendency for mice to remain close to walls when introduced into an open-field, serves as an index of anxiety-related behavior.

Two days after completion of the open-field testing, anxiety-related behavior was assessed using the elevated plus maze (EPM) according to previously described procedures [32] using a 1-day test. Eight-week-old male and female mice were evaluated over a 5-min period in an apparatus consisting of two opposing open arms (36 × 6.1 × 15 cm, clear Plexiglas) and two opposing enclosed arms (36 × 6.1 × 15 cm, black Plexiglas) extending from a central platform (5.5 × 5.5 cm) and equipped with computerized photobeam instrumentation (Kinder Scientific, Poway, CA). The 5-min test session began by placing a mouse in the center of the maze and allowing it to freely explore the apparatus. MotorMonitor software (Kinder Scientific, Poway, CA, USA) was used to record and analyze beam-break data to quantify time spent, distance traveled, and entries made into the open and closed arms and center area. Evaluating anxiety-related behavior in the EPM is based on the natural aversion of rodents to height and unfamiliar open spaces.

### 2.10 Statistics

Statistical comparisons were performed in GraphPad Prism. Statistical tests included unpaired t-test, 2-way ANOVA with Sidak's multiple comparisons test, mixed model, and log-rank tests. Individual statistical tests are detailed in the figure legends. Statistical significance was set at 0.05. For ANOVA and mixed model, significant (p < 0.05) and trending (p < 0.1) interaction terms are presented on the graphs (*e.g.* time x genotype). In the absence of a significant interaction by ANOVA, individual terms with associated p-values are displayed.

## 3 Results

### 3.1 Nav1.8-Cre targets peripheral sensory nerves, a subpopulation of autonomic neurons in the sympathetic chain, and several regions of the brain

**Figure 1:**
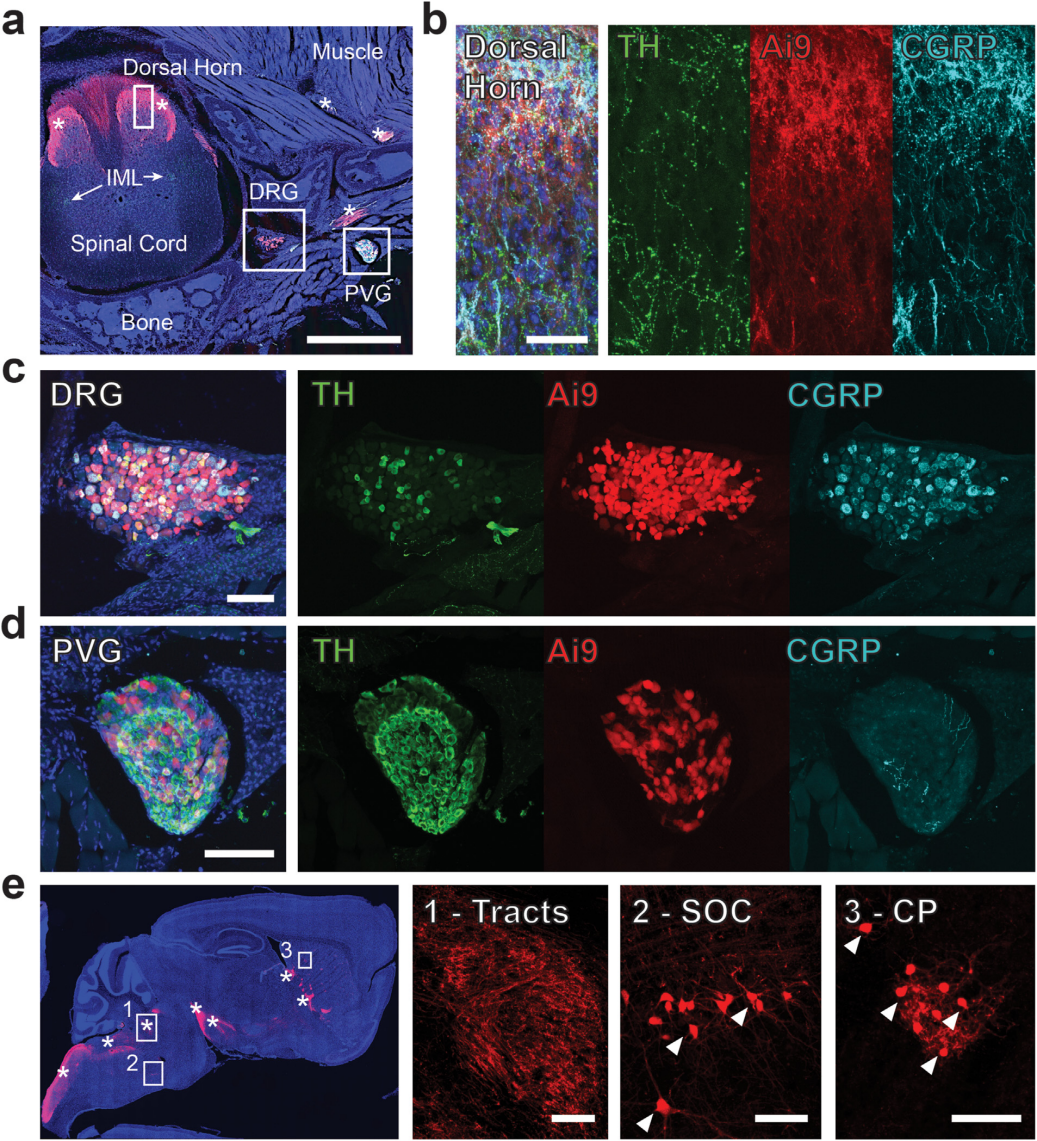
Nav1.8-Cre traces to sensory neurons, a subpopulation of postganglionic sympathetic neurons, and several regions of the brain. Nav1.8-Cre mice were bred with Rosa26-tdTomato (Ai9) reporter animals prior to cross-sectional analysis of the thoracic spine. After decalcification, 50 μm thick frozen sections containing the Ai9 reporter (red) were immunostained for tyrosine hydroxylase (TH, green) and calcitonin gene related peptide (CGRP, cyan) with DAPI as a nuclear counterstain (blue). **(a)** Representative section of the thoracic spine including a sensory dorsal root ganglia (DRG), a paravertebral ganglia within the sympathetic chain (PVG), and features of the spinal cord such as the dorsal horn and intermediolateral nucleus (IML). Scale = 500 μm. High-resolution images of the **(b)** dorsal horn, scale = 50 μm **(c)** DRG, scale = 100 μm and **(d)** PVG, scale = 100 μm showing the distribution of Nav1.8 traced neurons (Ai9+). **(e)** Sagittal cross-section of the brain showing (1) dorsal fiber tracts and labeled cell bodies in the (2) superior olivary complex (SOC) and (3) caudoputamen. Scale = 100 μm. Arrowheads = nerve cell bodies. ∗Axon fiber tracts. Representative images from adult mice at 10- to 12-weeks of age.

The Nav1.8-Cre mouse was generated in 2005 and is routinely used to selectively target the small diameter peripheral sensory neurons, including A and C fiber nociceptors [[21], [22], [23]]. To validate this for our study, we bred the Nav1.8-Cre mouse with Rosa26-ZsGreen (Ai6) or Rosa26-tdTomato (Ai9) reporter mice prior to analysis of the peripheral nervous system, brain, and common metabolic tissues in adult animals [28]. Consistent with prior reports, Nav1.8-Cre traced to peripheral sensory nerve cell bodies within the DRG and corresponding axons within the sensory laminae of the spinal cord (Figure 1A–C). This included populations of CGRP and TH positive nerves within the DRG (Figure 1B). In addition, Nav1.8-Cre traced a subpopulation of neurons within the paravertebral ganglia of the sympathetic chain (Figure 1D). By contrast, Nav1.8 tracing was not present in the pre-ganglionic autonomic axons within the intermediolateral nucleus of the spinal cord (Figure 1A).

In the brain, Nav1.8 traced axons projected through dorsal fiber tracts to several higher-order brain regions (Figure 1E and Supplemental Figure 1). Axonal labeling is expected based on the labeling of peripheral sensory neurons by Nav1.8-Cre, and the projection of these neurons to the brain. However, in addition to this, we observed regions of Nav1.8-Cre traced neural cell bodies in the superior olivary complex, hypothalamus, amygdala, bed nucleus of the stria terminalis, and caudoputamen (Figure 1E and Supplemental Figure 1). By contrast, labeling of cell bodies was absent in regions important for the sensory-discriminative aspects of nociception, including the parabrachial nucleus, thalamus, and somatosensory cortex.

In peripheral tissues, the Nav1.8-Cre reporter was restricted to axons within all tissues analyzed, including liver, kidney, pancreas, spleen, adrenal gland, adipose, muscle, ligament, bone, and associated vasculature and fascia (Figure 1 and Supplemental Figure 2). Overall, our results affirmed the targeted expression of Nav1.8 in peripheral neurons [21,23,33], which was most abundant in small sensory fibers. In addition, we also identified a subpopulation of postganglionic autonomic neurons within the thoracic sympathetic chain and localized populations of neurons within several brain regions that trace with Nav1.8-Cre.

### 3.2 Knockout of TSC2 with Nav1.8-Cre drives activation of mTORC1 pathways in peripheral sensory neurons

**Figure 2:**
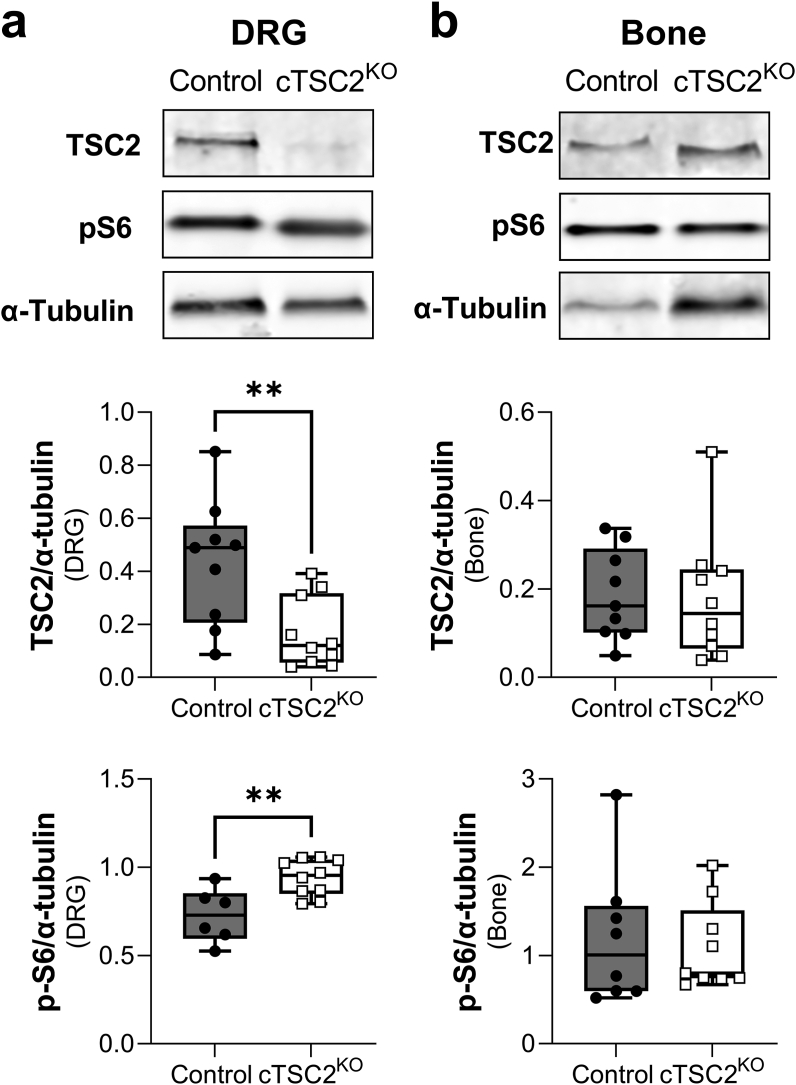
Validation of the conditional Nav1.8-TSC2^KO^ model. Western blots were used to quantify the depletion of TSC2 in control and conditional Nav1.8-TSC2^KO^ tissues at 12-weeks of age (cTSC2^KO^). We also quantified the phosphorylation of mTORC1 downstream target S6. Significant decreases in TSC2 and increases in p-S6 were present in Nav1.8-targeted sites such as dorsal root ganglia (DRG, a) but not in non-targeted tissues such as bone (right, b). Partial Nav1.8-Cre mediated depletion of TSC2 in the DRG is expected based on the presence of non-targeted cells such as glia, immune cells, etc. n = 6–10/group, male and female mice pooled. Presented as min to max box and whisker plot. Uncropped blots are available in Supplemental Fig. 3. Unpaired t-test. ∗∗p < 0.01.

To validate the Nav1.8-TSC2^KO^ model, we performed western blot analysis of TSC2 and p-S6, a downstream target of mTORC1, in whole DRG tissues. We repeated these analyses using bone as a negative control since it is not targeted by Nav1.8-Cre. We found that Nav1.8-TSC2^KO^ mice had a 60% decrease in TSC2 expression within the DRG (p = 0.007, Figure 2A and Supplemental Figure 3). We expect that the residual TSC2 expression detected within the Nav1.8-TSC2^KO^ DRG tissue is due to expression in the Nav1.8-negative sensory neurons and non-neural populations such as resident glial cells. Consistent with a prior report in a comparable model [5], knockout of TSC2 in Nav1.8+ sensory neurons corresponded to a 29% increase in p-S6 in the DRG (p = 0.004). There were no differences in TSC2 or p-S6 in bone tissues (negative control, Figure 2B and Supplemental Figure 3). This shows that TSC2 knockout was sufficient to enhance mTORC1 activation *in vivo* and that this was targeted to the Nav1.8-lineage + neurons. This is consistent with prior work in mice and the corresponding clinical condition in humans establishing that inactivation or knockout of TSC2 leads to maximal constitutive activation of mTORC1 [[4], [5], [6], [7]].

### 3.3 Nav1.8-TSC2^KO^ mice on control chow diet have mild, sex-specific reductions in body size in males but are otherwise comparable to controls

**Figure 3:**
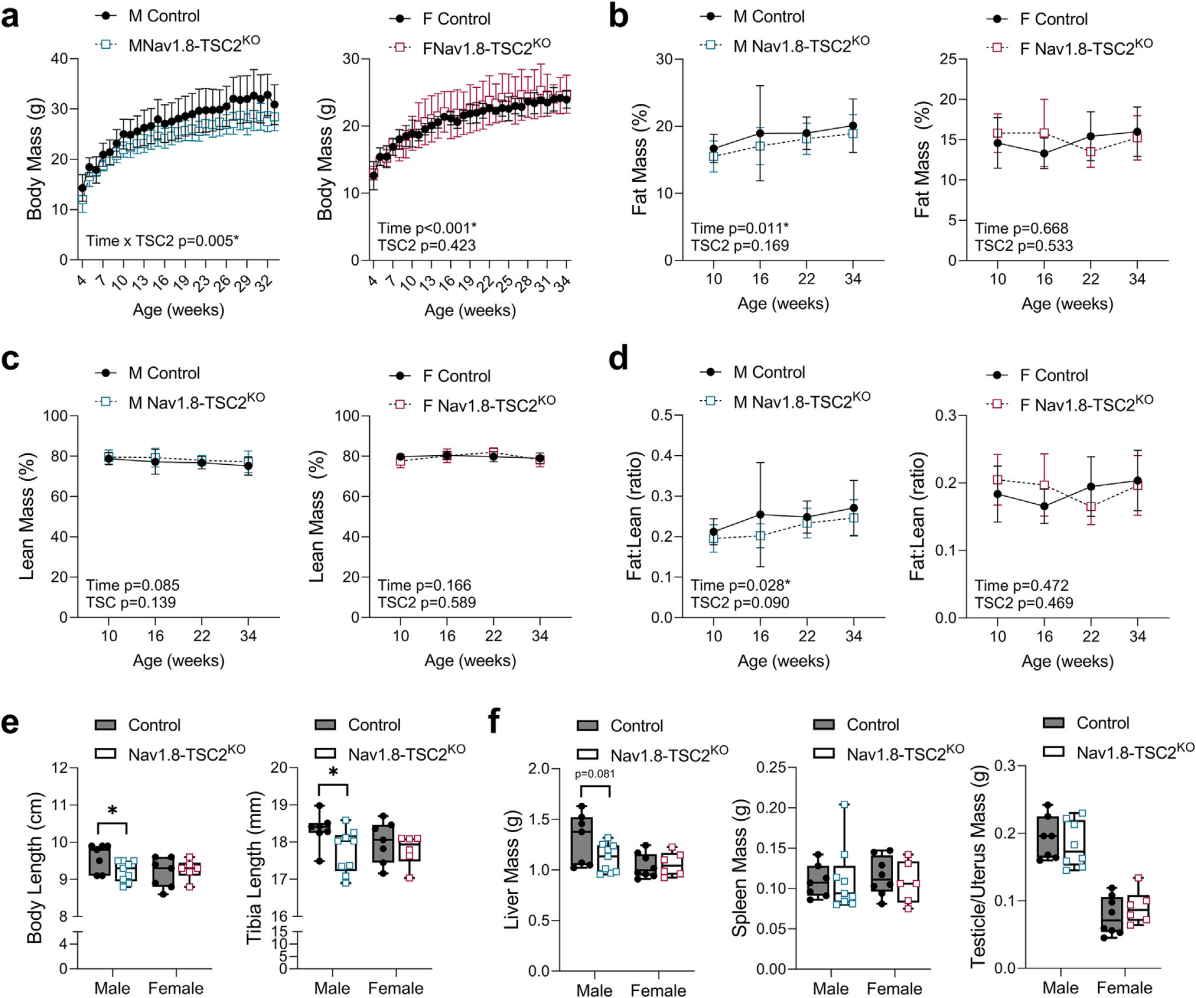
Nav1.8-*Tsc2*^KO^ mice have sex-specific decreases in body size with no changes in body composition. Representative data from male (M) and female (F) control and Nav1.8-TSC2^KO^ mice fed control chow diet. **(a)** Body mass from 4- to 34-weeks of age. **(b,c)** Fat and lean mass expressed as a proportion of total body mass as determined by EchoMRI at the indicated ages. **(d)** Ratio of fat to lean mass. **(e)** Body and tibia length measured at endpoint dissection at 10-weeks of age. **(f)** Post-mortem tissue mass measured at endpoint dissection at 10-weeks of age. ^a-d^ Mixed effects analysis (time x genotype). n = 4–21 mice per group per time point. Presented as mean ± SD. ^e,f^ Unpaired t-test between control and KO. Males and females analyzed independently. n = 6–9 as indicated on the graphs. Presented as min to max box and whisker plot. ∗p < 0.05.

The body mass of male Nav1.8-TSC2^KO^ mice on chow diet was 10.8% lower, on average, than control littermates from the earliest assessed time point at 4-weeks of age (Figure 3A). This difference persisted until the latest time point analyzed at 34-weeks of age. By contrast, the weight of female Nav1.8-TSC2^KO^ mice was comparable to controls (Figure 3A). Decreased body mass in the males was due to mild decreases in fat and lean mass that remained proportional to body mass, suggesting an overall minor decrease in body size (Figure 3B–D). Consistent with this, body and tibia length were decreased by 4% and 3%, respectively, in male but not female mice (Figure 3E). Males also had trending sex-specific decreases in liver tissue mass while spleen and gonad mass remained unchanged (Figure 3F). Altogether, multiple cohorts of animals confirmed consistent mild decreases in male, but not female, body size after knockout of TSC2 in Nav1.8+ neurons.

### 3.4 Nav1.8-TSC2^KO^ mice resist increases in body mass on HFD

**Figure 4:**
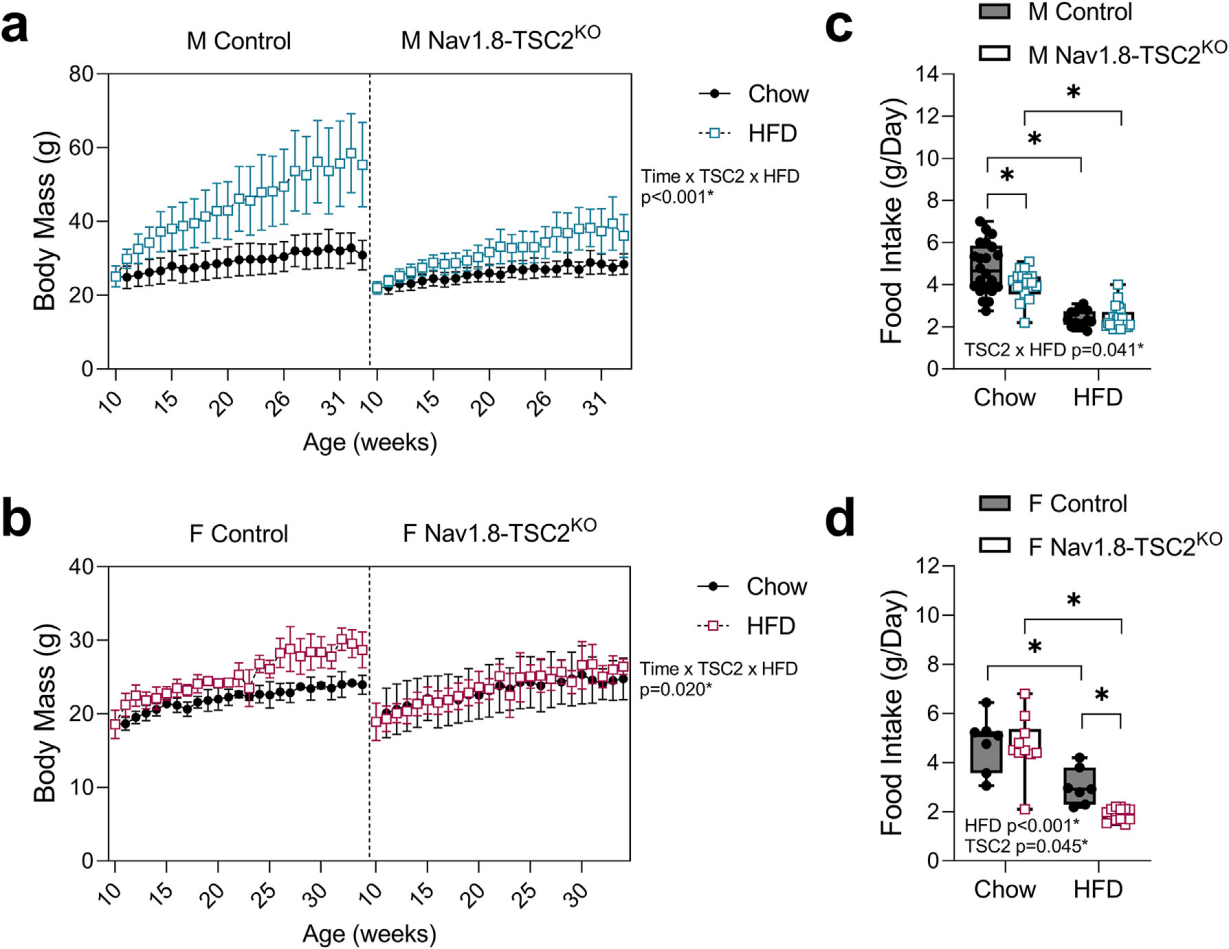
Nav1.8-TSC2^KO^ mice resist increases in body mass on high fat diet. Male (M) and female (F) control and Nav1.8-TSC2^KO^ mice were fed chow or 60% HFD for 24-weeks, from 10-to 34-weeks of age. **(a,b)** Body mass was measured on a weekly basis. **(c,d)** Food intake was measured weekly for the first 8-weeks of the study, from 10- to 18-weeks of age. ^a,b^ Mixed effects analysis (time x genotype x HFD). Male n = 8–10/group. Female n = 4–9/group. Presented as mean ± SD with 10-week baseline grouped prior to the start of HFD. ^c,d^ 2-way ANOVA with Tukey's multiple comparisons test. Presented as min to max box and whisker plot. ∗p < 0.05.

To isolate the capacity of neuronal TSC2 knockout to control the metabolic responses to HFD, both male and female Nav1.8-TSC2^KO^ mice and controls were fed standard chow or 60% HFD for 24-weeks, starting at 10-weeks of age. Male Nav1.8-TSC2^KO^ mice gained significantly less body mass on HFD and were, on average, 20–30% smaller than control mice on HFD (Figure 4A). Female Nav1.8-TSC2^KO^ mice also resisted increases in body mass with HFD (Figure 4B). At the end of the experiment, female control mice on HFD were 24% heavier than chow-fed controls while Nav1.8-Tsc2^KO^ mice were only 4% heavier than chow-fed Nav1.8-TSC2^KO^ mice. Overall, this experiment revealed that neural TSC2 knockout substantially decreased HFD-induced weight gain.

To explore the mechanism of reduced weight gain in the Nav1.8-TSC2^KO^ mice, we performed food intake assessments during the first 8-weeks of the HFD feeding period. Male Nav1.8-TSC2^KO^ mice on chow diet ate 16% less on average than controls (Figure 4C). However, when placed on HFD, the intake was the same as control mice. By contrast, female Nav1.8-TSC2^KO^ mice had a comparable intake on chow, but a 37% reduction when fed HFD (Figure 4D). Altogether, this suggests that while reduced food intake may contribute to the decreases in weight gain on HFD, particularly in females, it does not fully explain the phenotype in males.

### 3.5 Nav1.8-TSC2^KO^ mice develop obesity and impaired metabolic health, despite resistance to diet-induced weight gain (“normal weight obesity”)

**Figure 5:**
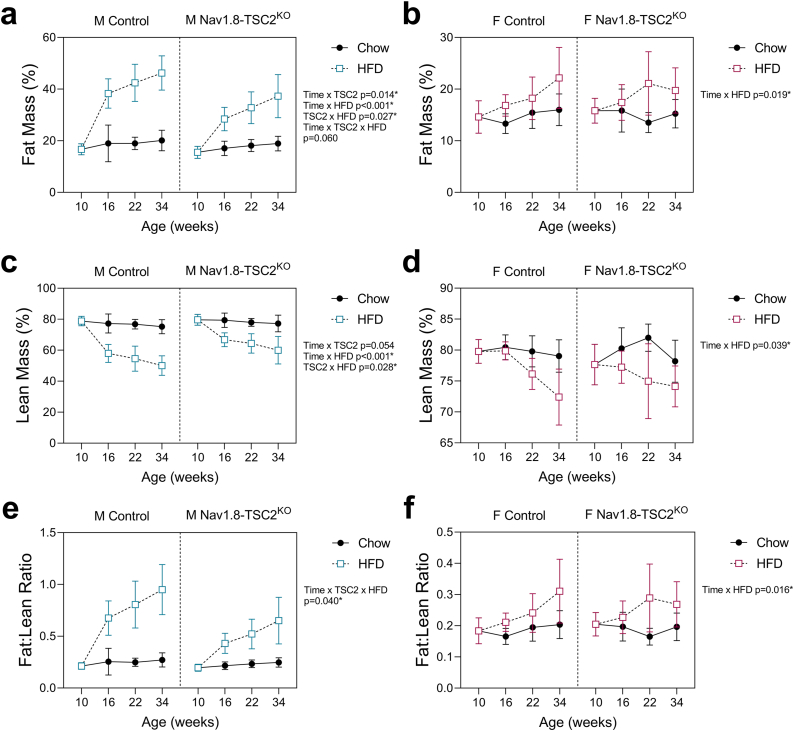
Both control and Nav1.8-TSC2^KO^ mice develop increased adiposity and increases in fat to lean mass when fed HFD. Male (M) and female (F) control and Nav1.8-TSC2^KO^ mice were fed chow or 60% HFD for 24-weeks, from 10-to 34-weeks of age. Body composition was measured by EchoMRI at 10-, 16-, 22-, and 34-weeks of age. **(a,b)** Fat mass as a % of body mass. **(c,d)** Lean mass as a % of body mass. **(e,f)** Fat mass to lean mass ratio. Mixed effects analysis (time x genotype x HFD). Male n = 8–10/group. Female n = 4–9/group. Presented as mean ± SD with 10-week baseline grouped prior to the start of HFD.

**Figure 6:**
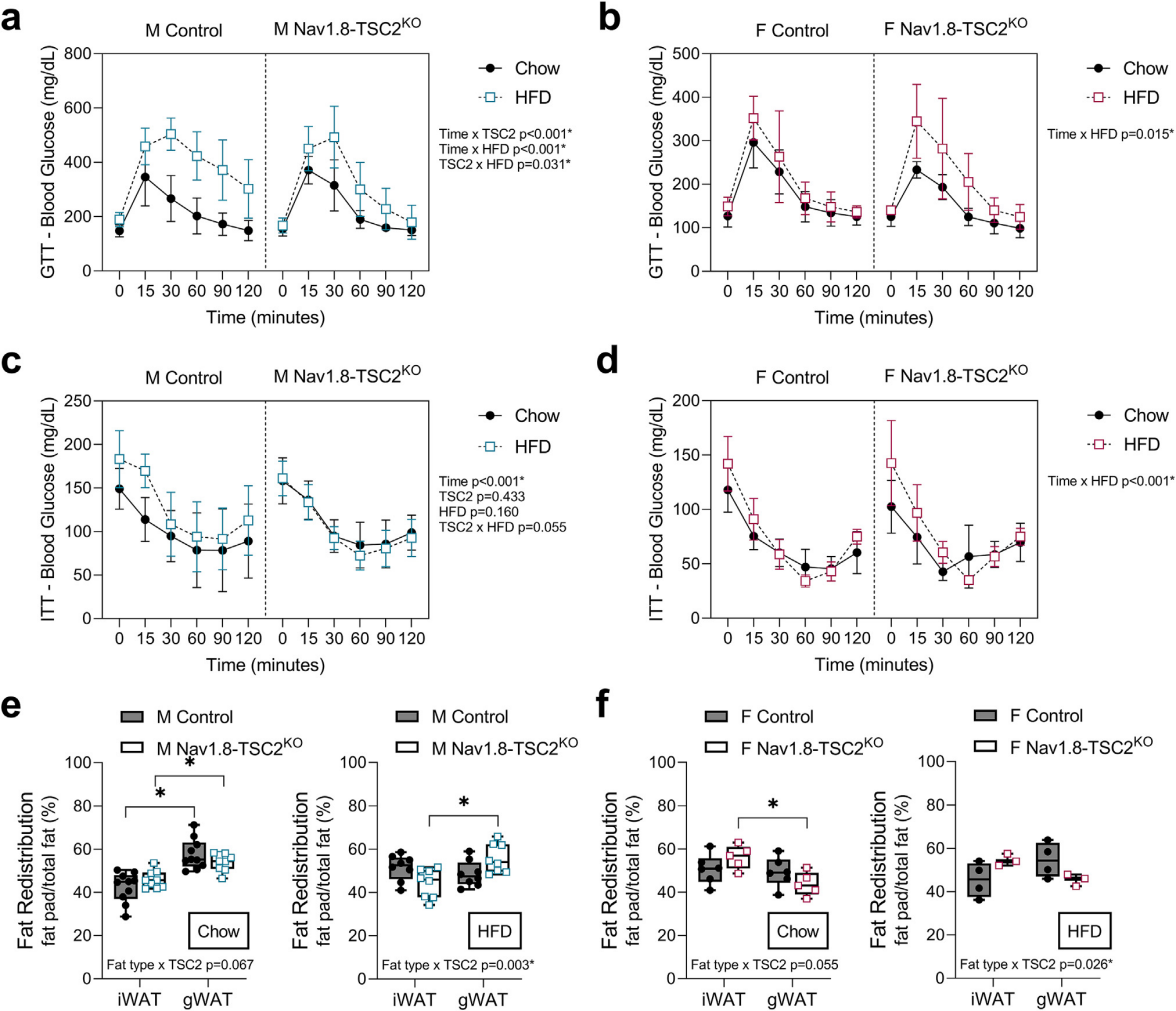
Both control and Nav1.8-TSC2^KO^ mice develop impaired metabolic health when fed HFD and have sex-specific alterations in fat distribution. Male (M) and female (F) control and Nav1.8-TSC2^KO^ mice were fed chow or 60% HFD for 24-weeks, from 10-to 34-weeks of age. Glucose and insulin tolerance tests were performed in the week prior to endpoint (GTT and ITT). **(a,b)** GTT results. **(c,d)** ITT results. **(e,f)** Gonadal and inguinal white adipose tissues (gWAT and iWAT) were dissected and weighed at endpoint after 24-weeks on either chow or HFD (age 34-weeks). Results expressed as the individual fat pad mass/total fat pad mass of iWAT + gWAT (%) to assess fat redistribution. Corresponding tissue mass values are available in Supplemental Fig. 5. ^a-d^ Mixed effects analysis (time x genotype x HFD). Male n = 8–10/group. Female n = 3–6/group. Presented as mean ± SD. ^e,f^ 2-way ANOVA with Tukey's multiple comparisons test (fat type x genotype). Presented as min to max box and whisker plot. ∗p < 0.05.

To quantify changes in adipose tissue and lean mass over time, we measured body composition by EchoMRI at the age of 10-, 16-, 22-, and 34-weeks (corresponding to 0-, 6-, 12-, and 24-weeks on chow or HFD). Despite resistance to gains in body mass (both males and females) and decreases in HFD food intake (females only), both male and female Nav1.8-Tsc2^KO^ mice exhibited significant increases in adiposity and percent body fat, consistent with the onset of obesity (Figure 5A,B; Supplemental Figure 4). This coincided with declines in percent lean mass and an increase in the fat-to-lean ratio by as early as 6-weeks after starting HFD (Figure 5C–F). To assess the metabolic health of the animals, we also performed glucose and insulin tolerance tests (GTT and ITT) in the week prior to endpoint. Both male and female mice exhibited HFD-dependent glucose intolerance, independent of genotype (Figure 6A,B). We did not observe overt insulin resistance during the time course of our experiment (Figure 6C,D). However, there were slight modifications to the insulin-dependent glucose response in females that were comparable in both HFD-fed control and Nav1.8-Tsc2^KO^ mice (Figure 6D). The Nav1.8-Tsc2^KO^ phenotype of adipose tissue expansion with impaired metabolic health, despite resistance to gains in body mass, mimics the clinical condition of normal weight obesity (also known as thin fat obesity, metabolic obesity, skinny fat, and metabolically unhealthy non-obese).

### 3.6 Nav1.8-TSC2^KO^ mice have sex-specific alterations in fat distribution

Peripheral nerves play an important role in maintaining fat distribution throughout the body [34,35]. Given this, we next examined if TSC2 knockout in the Nav1.8+ neurons was sufficient to modify the distribution of adipose tissues. Inguinal subcutaneous and gonadal visceral white adipose tissues (iWAT and gWAT) were weighed in a subset of mice at the time of endpoint dissection at 34-weeks of age, after the mice had been on chow or on HFD between the ages of 10- and 34-weeks (Supplemental Figure 5). Tissue weights were used to assess the distribution of fat between subcutaneous and visceral depots (fat pad mass/total fat with total being the sum of iWAT and gWAT). In male Nav1.8-TSC2^KO^ mice on chow diet, the distribution of fat between subcutaneous and visceral gonadal depots remained comparable to controls (Figure 6E). However, when placed on HFD, male Nav1.8-TSC2^KO^ mice displayed increased deposition of visceral fat as gWAT (Figure 6E). By contrast, female Nav1.8-TSC2^KO^ mice preferentially expanded their subcutaneous fat depots regardless of diet (Figure 6F). This unique depot-specific phenotype of adipose tissue expansion in the Nav1.8-TSC2^KO^ mice mirrors findings in human obesity whereby women often have a tendency to store fat in subcutaneous locations while men are often predisposed to visceral fat accumulation [36,37].

### 3.7 Nav1.8-TSC2^KO^ mice develop skeletal complications of HFD feeding, despite resistance to weight gain

**Figure 7:**
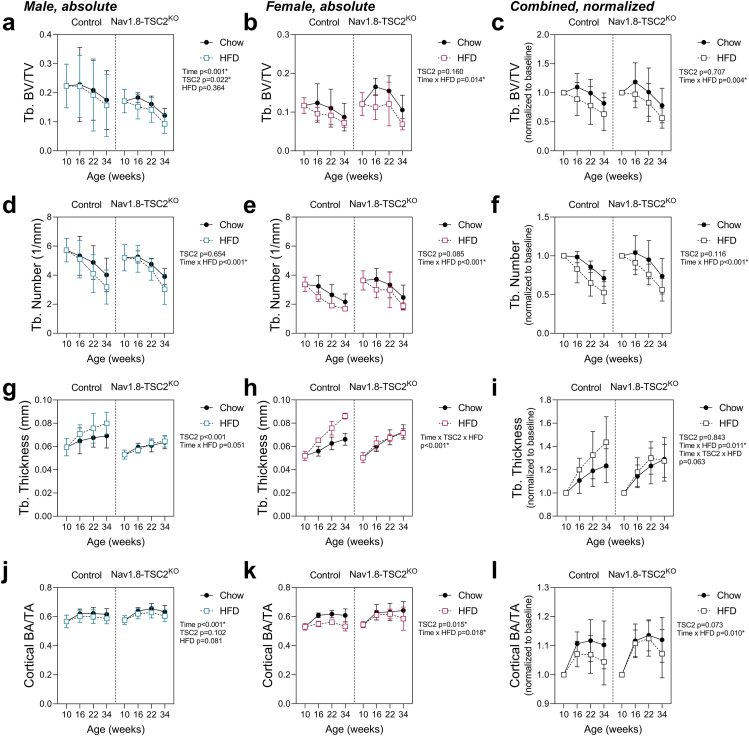
Both control and Nav1.8-TSC2^KO^ mice develop impaired bone mass when fed HFD, despite differences in body mass. Male (M) and female (F) control and Nav1.8-TSC2^KO^ mice were fed chow or 60% HFD for 24-weeks, from 10-to 34-weeks of age. Bone morphology was measured by vivaCT at 10-, 16-, 22-, and 34-weeks of age. **(a**–**c)** Trabecular bone volume fraction. **(d–f)** Trabecular number. **(g–i)** Trabecular thickness. **(j–l)** Cortical bone area per total area. Mixed effects analysis (time x genotype x HFD). Male n = 8–10/group. Female n = 4–9/group. Presented as mean ± SD with 10-week baseline grouped prior to the start of HFD.

Bone loss is a common secondary complication of HFD-associated metabolic disease [38,39]. To assess this, we quantified changes in bone microarchitecture by serial micro-computed tomography at 10-, 16-, 22-, and 34-weeks of age (Figure 7). Both control and Nav1.8-Tsc2^KO^ mice developed HFD-induced decreases in trabecular bone volume fraction (BVF) (Figure 7A–C). Loss of BVF was primarily due to decreases in trabecular number (Figure 7D–F). By contrast, HFD promoted gains in trabecular thickness in control, but not in Nav1.8-Tsc2^KO^ mice (Figure 7G–I). This may be due to the increased body mass of the HFD-fed control but not Nav1.8-Tsc2^KO^ animals, leading to differences in skeletal loading. As for trabecular BVF, HFD also decreased cortical bone area fraction independent of genotype (Figure 7J-l). Overall, these results reveal that despite resistance to weight gain, Nav1.8-Tsc2^KO^ mice experienced significant HFD-induced bone loss. In addition, these mice also lacked any benefits to bone from increased loading, resulting in further impairments to bone microarchitecture.

### 3.8 Nav1.8-TSC2^KO^ mice display evidence of chronic itch and anxiety

**Figure 8:**
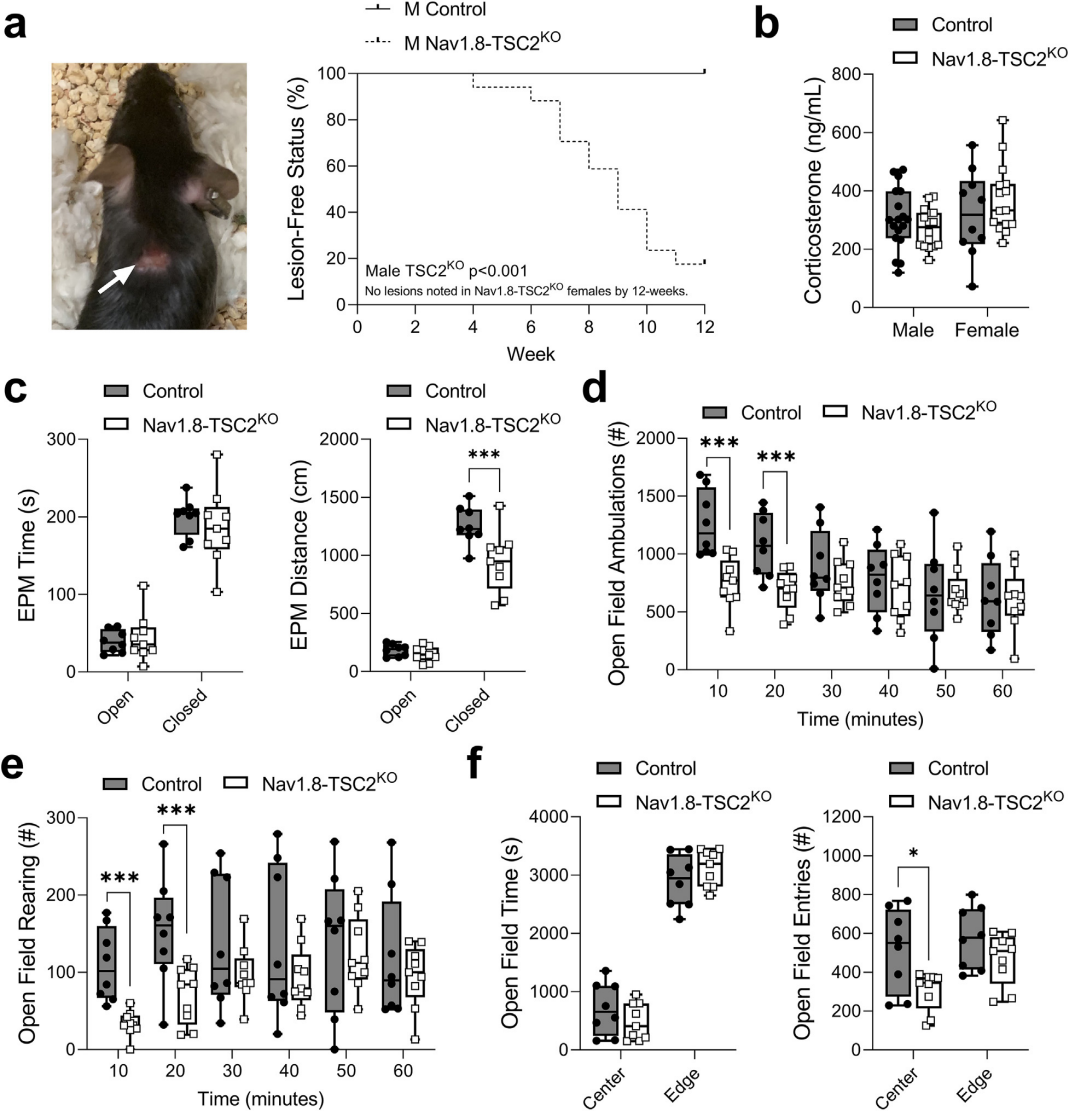
Nav1.8-TSC2^KO^ mice display evidence of chronic itch and anxiety-like behaviors. **(a)** Development of cutaneous lesions (arrow) localized to the neck and ears during the first 12-weeks of life in standard-chow fed males and females (n = 9–17/group). Log-rank test. **(b)** Plasma corticosterone measured between ZT7 and ZT10 after a 4-hour fast at 8- to 10-weeks of age from mice fed standard chow. **(c)** Elevated plus maze performed at 8-weeks of age in mice fed standard chow (n = 3 male/5 female control and 5 male/5 female Nav1.8-TSC2^KO^), time spent and distance traveled in the open vs closed arms. **(d,e)** Total ambulations and rearing as measured by open field assessment over a 60-minute period in 8-week old control and Nav1.8-TSC2^KO^ fed standard chow (n = 3 male/5 female control and 5 male/5 female Nav1.8-TSC2^KO^). **(f)** Grouped parameters of the open field including total time and number of entries in the center vs edge regions. (c–f) Males and females combined. ^b-f^ 2-way ANOVA (genotype x target variable) with Sidak's multiple comparisons test. Presented as min to max box and whisker plot. ∗p < 0.05, ∗∗∗p < 0.001.

Nav1.8-Tsc2^KO^ mice may have altered sensory or autonomic functions that mediate the HFD-induced onset of normal weight obesity. Prior work in a comparable Nav1.8-TSC2^KO^ model found that these mice have normal or slightly attenuated nociception when challenged with a battery of assessments including heat, cold, and mechanical stimuli [5]. Sensorimotor behavior was also comparable to controls by rotarod and pole climb [5]. Beyond this, during our initial experiments with the Nav1.8-TSC2^KO^ mice, we observed that some animals developed skin lesions, most notably around the craniofacial, dorsal neck, and interscapular regions (Figure 8A). In developing animals on control chow diet, this occurred in 82% of male and 0% of female Nav1.8-TSC2^KO^ mice by 12-weeks of age (Figure 8A). Though not quantified, many females went on to develop lesions at older ages (personal observation). This suggests that, though the timing of onset may be different, this phenotype is not sex-dependent. The pattern and progression of these lesions were consistent with previous studies of spontaneous chronic itch [40].

To consider the role of stress and anxiety in the normal weight obesity phenotype, we next sought to quantify any differences in the circulating stress biomarker corticosterone and in anxiety-like behaviors by elevated plus maze and open field. For the corticosterone assay, plasma was collected between ZT7 and ZT10 to capture the peak in circadian plasma corticosterone. We did not observe any differences in plasma corticosterone in Nav1.8-TSC2^KO^ mice at 8- to 10-weeks of age (Figure 8B). There were also no differences in the time spent or number of entries into the open and closed arms of the elevated plus maze at 8-weeks of age (Figure 8C and Supplemental Figure 6). When in the closed arm, however, the Nav1.8-TSC2^KO^ mice moved less than controls (Figure 8C). In the open field assessment, Nav1.8-TSC2^KO^ mice exhibited decreased exploration when placed into the new environment, as recorded by changes in ambulation and rearing within the first 20-minutes of the assessment (Figure 8D,E and Supplemental Figure 6). Nav1.8-TSC2^KO^ mice also had a decreased number of entries into the center region during the 60-minute open field assessment (Figure 8F). Overall, these results indicate that Nav1.8-TSC2^KO^ mice have behaviors consistent with chronic itch and mild increases in anxiety.

## 4 Discussion

### 4.1 The Nav1.8-TSC2^KO^ mouse is a new genetic model of normal weight obesity

Normal weight obesity (also known as thin fat obesity, metabolic obesity, skinny fat, and metabolically unhealthy non-obese) is defined as having normal weight and/or body mass index (BMI) with a high percentage of body fat, generally greater than 30% [41,42]. Current studies reveal that the worldwide prevalence of normal weight obesity ranges from 4.5 to 22% [41,42]. The mechanisms underlying the onset of normal weight obesity remain unresolved, with a multitude of conflicting clinical studies in the literature pertaining to the role of maternal health, race, physical activity, and diet (summarized in [41]). This likely indicates that there are multiple forms of normal weight obesity with divergent genetic and environmental risk factors, and potentially disparate consequences for systemic health. Initial studies have identified genetic polymorphisms in IL-1 receptor antagonist, IL-15 receptor alpha, MTHFR, and apolipoprotein E as related to normal weight obesity [[43], [44], [45]]. However, these studies have been relatively small and mostly limited to Caucasian females. Larger studies across sexes and racial groups are needed to clarify the genetic risk factors that predispose to the onset of normal weight obesity.

Beyond work in humans, the lack of defined rodent models further limits our identification of the underlying mechanisms that predispose to normal weight obesity. At the time of this publication, there have been only two prior reports of normal weight obesity in rodents [46,47]. A 2016 review of both normal weight obesity and the converse phenotype of metabolically healthy obese (MHO) identified several mouse models of MHO with none for normal weight obesity [48]. Furthermore, the concept of normal weight obesity is generally not addressed or recognized in existing reviews of the preclinical models for obesity research [49]. With increased awareness, it is likely that new genetic risk factors and mechanisms of normal weight obesity will emerge in the coming decades. This work uncovers TSC2 loss of function in Nav1.8-lineage + neurons as a novel mechanism driving the diet-dependent onset of this unique phenotype.

### 4.2 Normal weight obesity in Nav1.8-TSC2^KO^ mice masks underlying changes in metabolic and bone health

The main clinical concern with normal weight obesity is that the ‘normal’ BMI of these individuals masks underlying adipose tissue expansion and is associated with a high prevalence of diabetes, hypertension, and dyslipidemia with an increase in all-cause mortality [42]. In our work, we observed similar results. Though Nav1.8-TSC2^KO^ mice resisted weight gain on HFD, they developed comparable adipose tissue expansion and metabolic disease. This mirrors the onset of type 2 diabetes in humans. In addition to this, we found that HFD-fed Nav1.8-TSC2^KO^ mice developed bone loss and changes in microarchitecture that were even more severe than HFD-fed controls. This may be due to the onset of metabolic disease with loss of the protective effects of excess body weight on skeletal loading and bone anabolic responses. This represents an important area of future intervention because, in older adults, ∼8–20% die within 1-year of hip fracture, and >50% never regain functional independence [[50], [51], [52]]. These outcomes are even worse for patients with diabetes [[53], [54], [55], [56], [57], [58]].

### 4.3 Lineage tracing with Nav1.8-Cre reveals targeted neuronal populations both in the periphery and in the brain

The work in this paper focused on the effects of TSC2 deletion in the Nav1.8-Cre+ neural lineage. Nav1.8, also known as SNS or PN3, was originally cloned and identified as a sensory nerve specific, tetrodotoxin-resistant voltage-gated sodium channel by two groups in 1996 [33,59]. Since then, Nav1.8 has become well established as a marker of peripheral nociceptors and Nav1.8-Cre has been routinely used to target small diameter sensory nerves [60]. Our lineage tracing results with Nav1.8-Cre confirmed this expression. However, we also identified a relatively high number of traced neurons in the paravertebral sympathetic ganglia. This is similar to two prior reports of Nav1.8-Cre lineage tracing that identified a small population of Nav1.8+ cells in the autonomic superior cervical ganglion [21,23]. Our co-immunostaining also identified overlap of Nav1.8+ sensory neurons with both CGRP and TH. Within the neuronal populations in the DRG, CGRP is expressed by sensory peptidergic neurons, and TH is expressed by a unique class of low-threshold sensory mechanoreceptors [61]. Our results are consistent with prior reports showing that Nav1.8-Cre targets >90% of neurons expressing markers of nociceptors in addition to at least two types of low-threshold mechanoreceptors [22].

In addition to peripheral nerves, we also identified several regions in the brain that were targeted by Nav1.8-Cre. This conflicts with a previous study using the same Nav1.8-Cre model, though with a different β-galactosidase-based reporter assay, which failed to detect Nav1.8+ neurons in the brain or spinal cord [23]. The difference here is likely due to the increased sensitivity of our reporter system. Consistent with this, a separate study that linked EGFP to a 3.7 kb putative promoter of Nav1.8 identified expression in regions of the limbic system including the amygdala, globus pallidus, and hypothalamus, as well as regions of sensory processing including the olfactory tubercle and somatosensory cortex [62]. Similar limbic regions were observed from WGA tracing of Nav1.8+ nociceptors including the hypothalamus, amygdala, and the bed nucleus of the stria terminalis, but few labeled regions were associated with the sensory-discriminative aspects of nociception [63]. These results are comparable to our observations with the Ai9 reporter. The implications of this finding remain unclear but suggest that prior results obtained with the Nav1.8-Cre model that were attributed solely to the actions of peripheral nerves may need to be re-examined. This also supports the future investigation of Nav1.8-Cre targeted neurons in the brain.

### 4.4 Contributions of TSC2 knockout in Nav1.8-Cre+ neurons to chronic itch and anxiety

In this study, apart from minor decreases in body size in males, the Nav1.8-TSC2^KO^ mice were generally indistinguishable from controls. The one exception to this was the gradual onset of localized skin lesions with an appearance consistent with chronic itch. Prior studies have shown that expression of a constitutively active form of BRAF in Nav1.8+ neurons results in a chronic itch phenotype that has a similar presentation and time of onset to our Nav1.8-TSC2^KO^ mice [40]. Chronic mTORC1 activation in A-fibers has also been implicated in the pathogenesis of itch and ∼40% of A-fibers are targeted by Nav1.8-Cre [22,66]. This suggests a direct contribution of mTORC1 activation in the Nav1.8-Cre targeted peripheral nerves to the onset of the itch phenotype in our model. In addition to itch, the Nav1.8-TSC2^KO^ mice had an early onset of anxiety-like behaviors. In humans, chronic itch and anxiety are closely linked and can set up a vicious cycle of anxiety exacerbating itch and itch contributing to anxiety. However, it is unclear if the two are related in our model, or if the anxiety phenotype is a result of the actions of Nav1.8-Cre within the CNS.

### 4.5 Neuronal function as a determinant of sex-specific adipose tissue distribution

As a final component of our study, we considered a putative neural basis for adipose tissue distribution in our model. We found that Nav1.8-TSC2^KO^ males preferentially deposited visceral gonadal white adipose tissue when on HFD. By contrast, female Nav1.8-TSC2^KO^ mice stored increased subcutaneous fat regardless of diet. This mirrors sex-specific differences in humans whereby males are often more susceptible to visceral adipose tissue accumulation while it is common for females to preferentially store subcutaneous fat [36,37]. Our work reinforces the concept that underlying differences in the function of the nervous system may drive sex-specific variations in adipose tissue distribution [34,35].

### 4.6 Possible mechanisms of normal weight obesity in Nav1.8-TSC2^KO^ mice

This study identified upregulated neural mTOR signaling through TSC2 knockout as a key mechanism that predisposes to the HFD-associated onset of normal weight obesity. Beyond this, the possible mechanisms underlying this phenotype are multifold. Vagal afferents, important for feeding and metabolic regulation, have been shown to contain populations of Nav1.8+ neurons [21,67]. Constitutive activation of mTOR in these neurons may result in altered signaling downstream of metabolic hormones like leptin, impacting food intake, fat storage and metabolism. In the brain, Nav1.8+ neurons were observed in sites established to regulate metabolic activity including the lateral hypothalamus, a vital region in the regulation of metabolism and sensation of hunger. We also observed Nav1.8-Cre targeted connections in the brain that were localized to regions of affective/emotional function. This may adjust mood or motivation in response to aberrant signaling. For instance, changes in the activity in the bed nucleus of the stria terminalis could evoke pathological anxiety [64] and pathways connecting the amygdala and the globus pallidus could modulate behavior in response to perceived frightful stimuli [65]. Though we did not detect gross elevations in corticosterone, slight chronic increases due to ongoing stress could lead to increased fat deposition, at the expense of muscle and bone mass [68,69]. Additional models will be required to differentiate the effects of upregulated mTOR signaling in central *vs* peripheral Nav1.8 lineage neurons on altering affect and metabolism and their consequences for the onset of the normal weight obesity phenotype.

## 5 Conclusions

This study reveals that knockout of TSC2 in Nav1.8+ neurons increases itch- and anxiety-like behaviors and substantially modifies the distribution of adipose tissues and the metabolic responses to HFD. Though it prevents HFD-induced weight gain, this masks persistent detrimental effects on metabolic health and peripheral organs such as bone, mimicking the ‘normal weight obesity’ phenotype that is of growing concern. This work supports a mechanism by which targeted increases in neuronal mTOR signaling predispose to altered adipose tissue distribution, adipose tissue expansion, impaired peripheral metabolism, and detrimental changes to skeletal health with HFD – despite resistance to diet-induced weight gain.

## Conflict of interest

The authors have no conflict of interest to declare.

## Data Availability

Data will be made available on request.
