# Supplementary material for: Knockout of TSC2 in Nav1.8+ neurons predisposes to the onset of normal weight obesity
Source: Mol Metab. 2022 Dec 28;68:101664. doi: 10.1016/j.molmet.2022.101664 (PMC9841058; doi:10.1016/j.molmet.2022.101664)
Supplement: Multimedia component 1 [file mmc1.docx]

**SUPPLEMENTAL FIGURE 1**


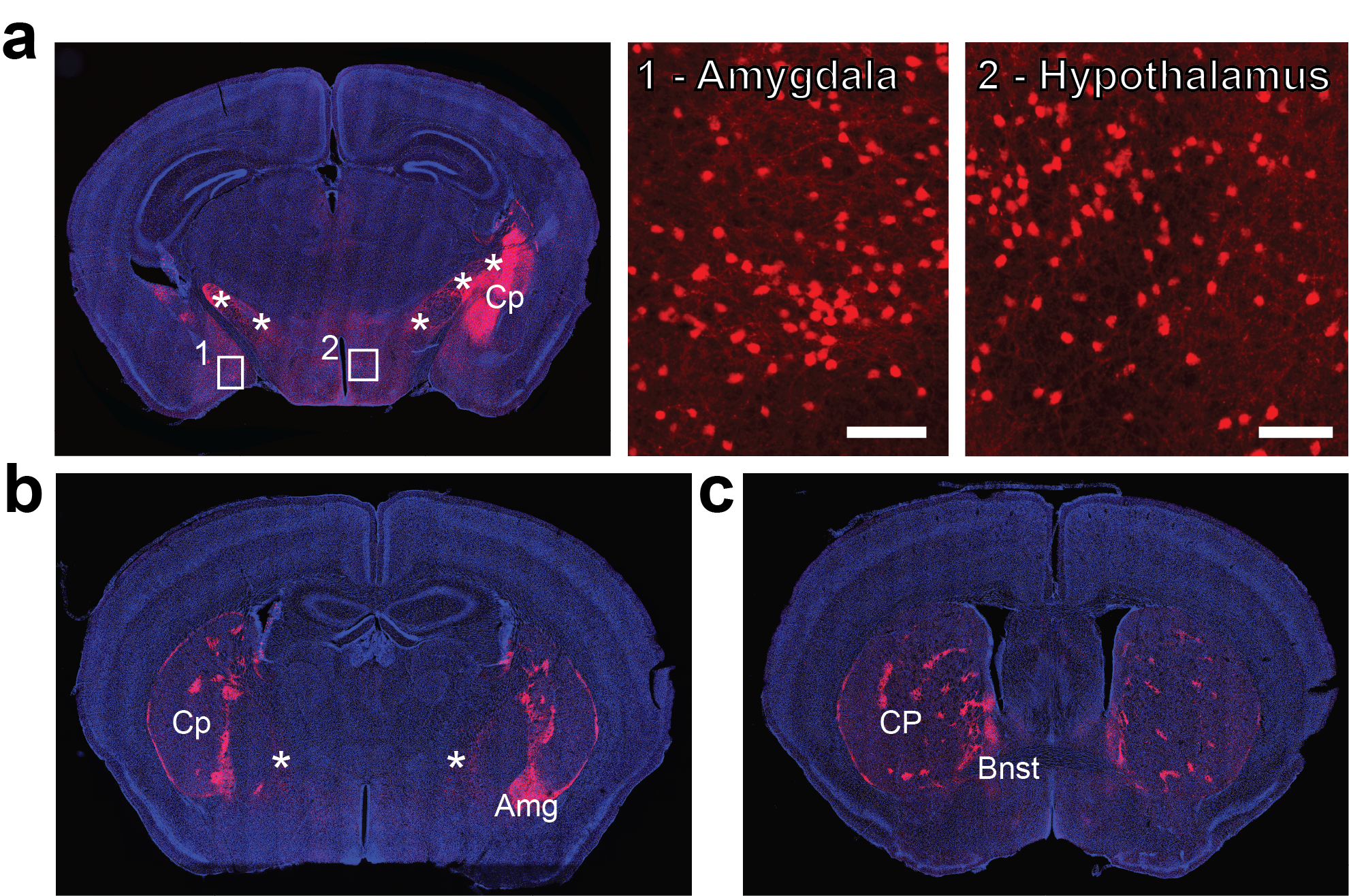


**Supplemental Figure 1. Nav1.8-Cre traces to several regions of the brain.** **(a,b)** Representative midbrain sections showing lineage traced Ai9-expressing neurons (red) in the amygdala (Amg), hypothalamus, and caudoputamen (Cp). **(c)** Representative forebrain section showing traced Ai9-expressing neurons in the Cp and the bed nucleus of the stria terminalis (Bnst). Blue = DAPI. *Axon fiber tracts. All scale bars=100 µm.

**SUPPLEMENTAL FIGURE 2**


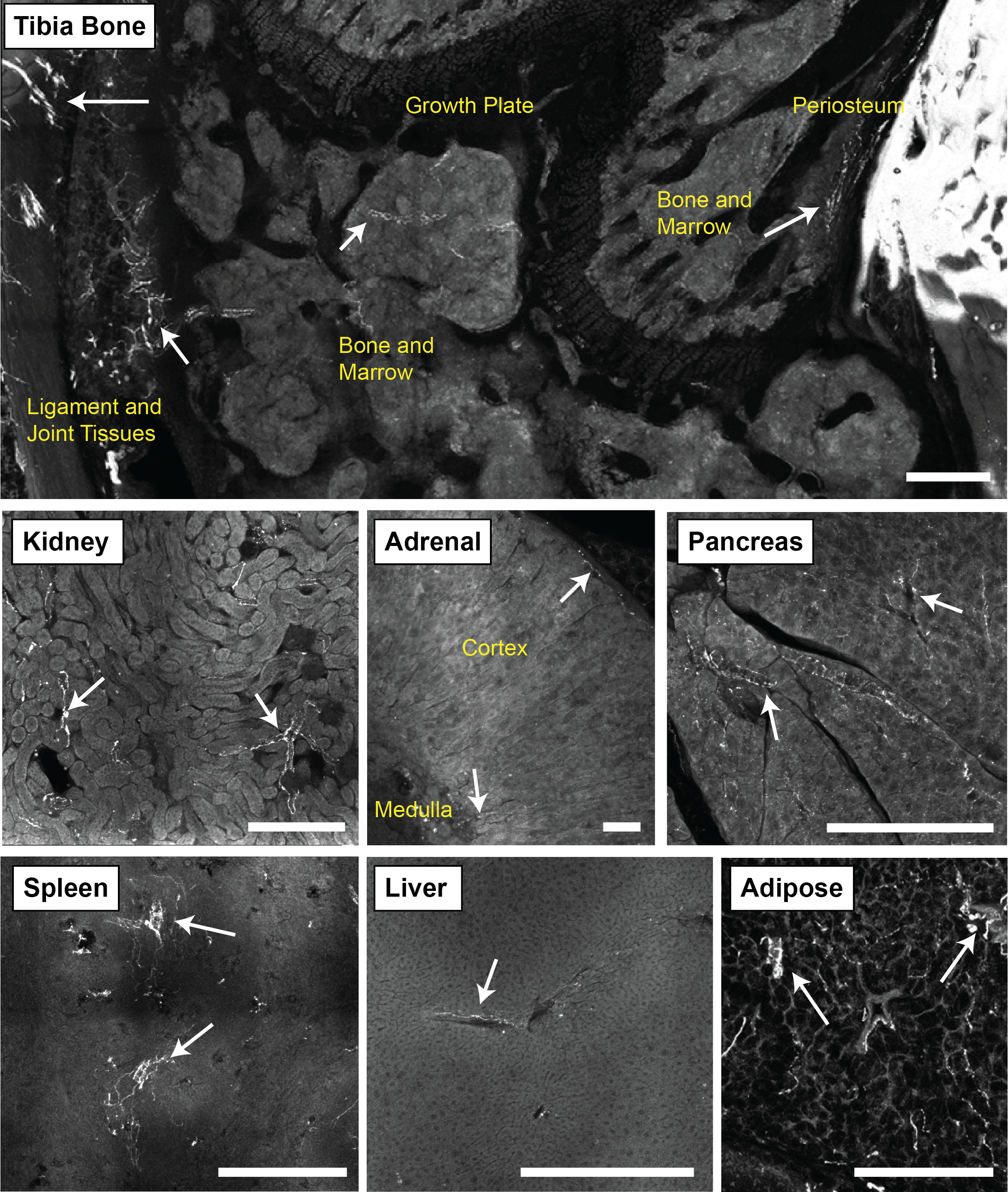


**Supplemental Figure 2. Nav1.8-Cre traces to peripheral nerves (only) in a diverse array of tissues.** Nav1.8-Cre mice were bred with Rosa26-ZsGreen (Ai6) reporter animals prior to cross-sectional analysis of an array of tissues. Results presented in greyscale (Ai6 reporter = white). Tissue names as indicated in the figures. Nav1.8-Cre traced axons in all peripheral tissues examined (arrows). We did not observe any evidence of non-neuronal labeling of the Nav1.8 reporter. All scale bars=250 µm.

**SUPPLEMENTAL FIGURE 3**

**
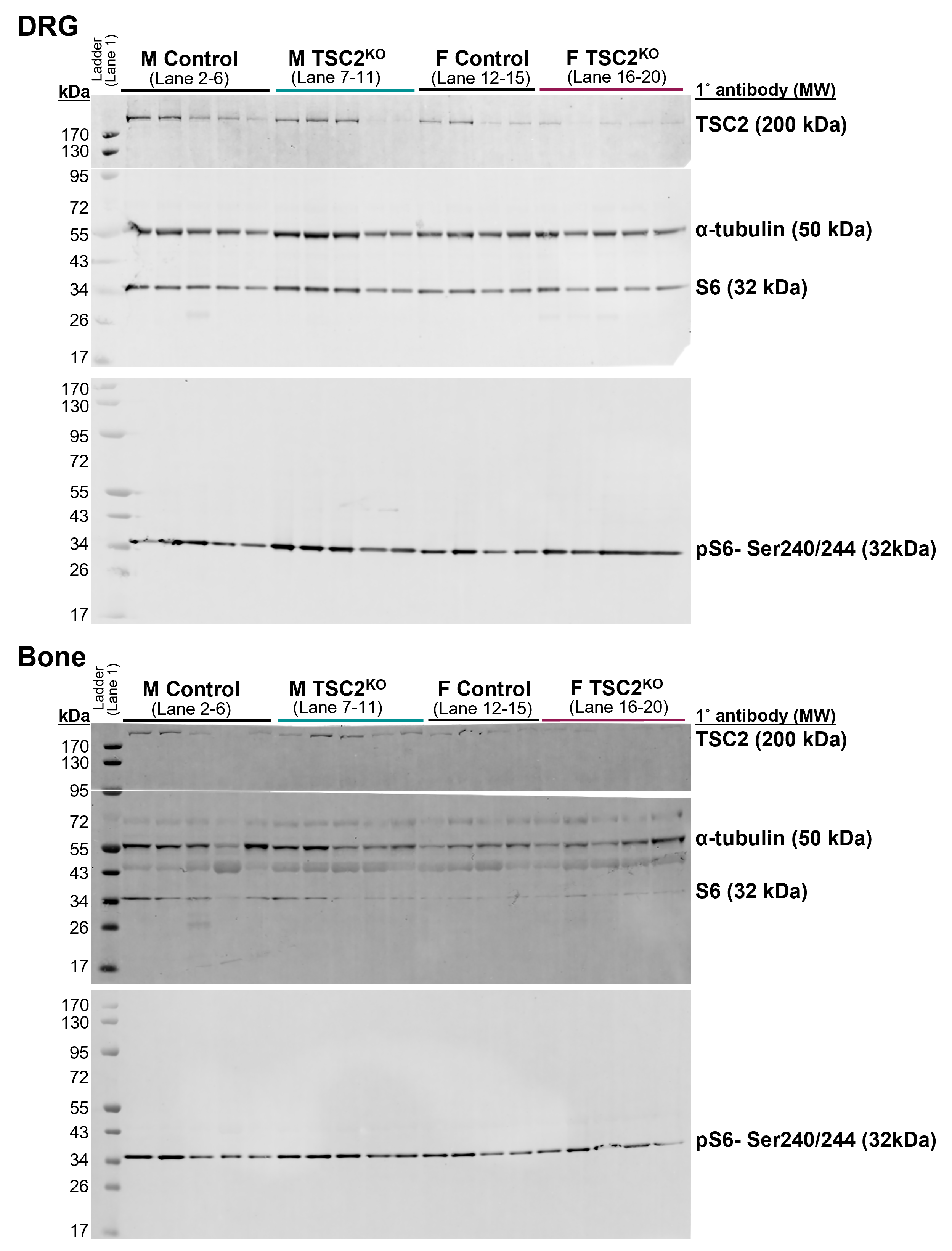
**

**Supplemental Figure 3. Uncropped western blots.** Western blots were used to quantify the depletion of TSC2 in control and conditional Nav1.8-TSC2^KO^ animals at 12-weeks of age (cTSC2^KO^). We also quantified the phosphorylation of mTORC1 downstream target S6.

**SUPPLEMENTAL FIGURE 4**

**
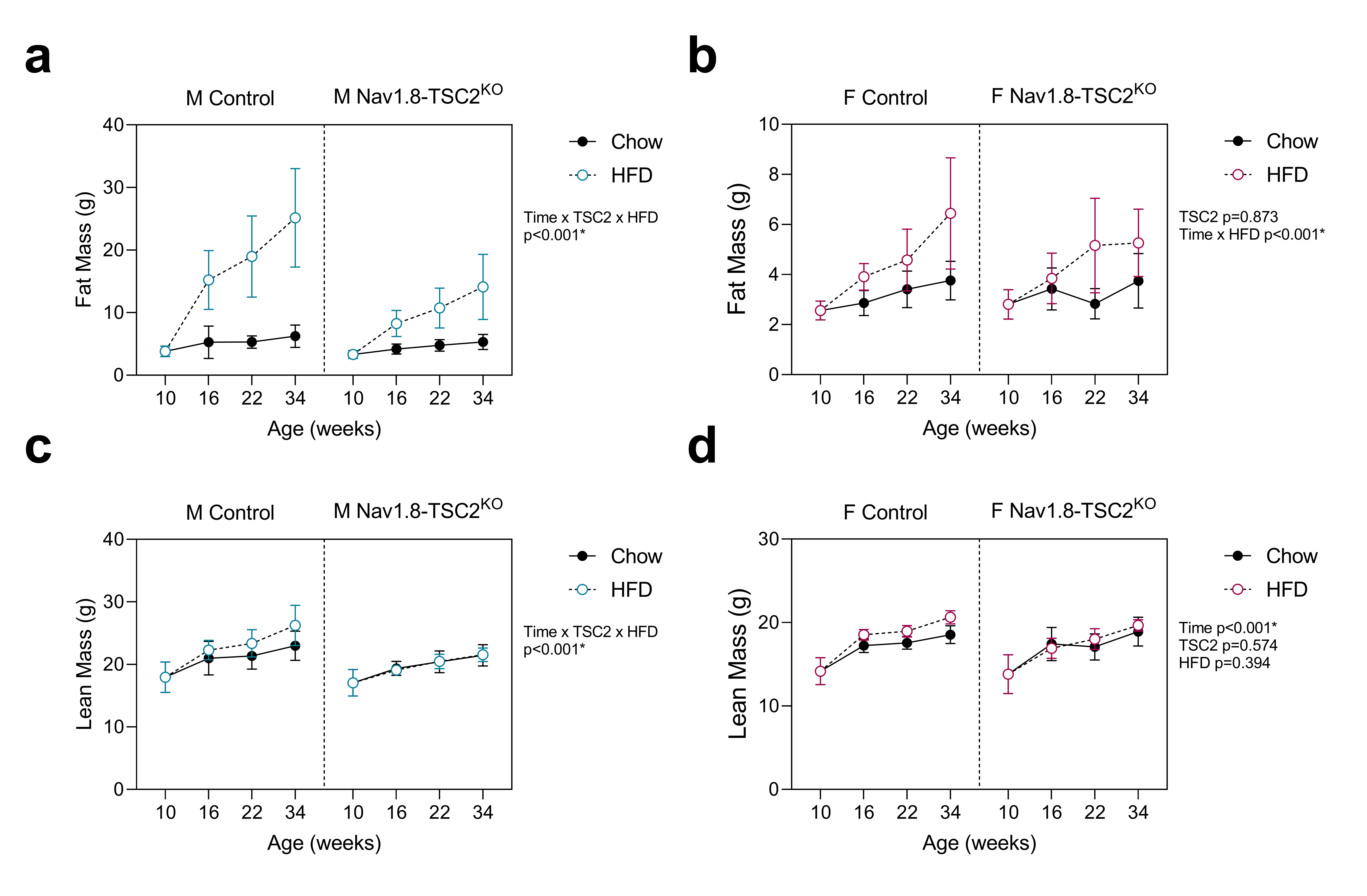
**

**Supplemental Figure 4. Nav1.8-TSC2^KO^ mice have evidence of increased fat mass and suppression of lean mass accrual when on HFD (“normal weight obesity”).** Male (M) and female (F) control and Nav1.8-TSC2KO mice were fed chow or 60% HFD for 24-weeks, from 10-to 34-weeks of age. Body composition was measured by EchoMRI at 10-, 16-, 22-, and 34-weeks of age. **(a,b)** Absolute fat mass. **(c,d)** Absolute lean mass. Mixed effects analysis (time x genotype x HFD). Male n=8-10/group. Female n=4-9/group. Presented as mean$\pm$SD with 10-week baseline grouped prior to the start of HFD.

**SUPPLEMENTAL FIGURE 5**





**Supplemental Figure 5. Changes in fat pad mass with HFD in control and Nav1.8-TSC2^KO^ mice.** Male (M) and female (F) control and Nav1.8-TSC2^KO^ mice were fed chow or 60% HFD for 24-weeks, from 10-to 34-weeks of age. In a subset of animals, the gonadal and inguinal white adipose tissues (gWAT and iWAT) were dissected and weighed at end point (mouse age 34-weeks). Results are expressed as the absolute tissue mass in grams (g) and the % of total body mass. (a,b) iWAT mass. (c,d) gWAT mass. 2-way ANOVA with Tukey’s multiple comparisons test. Presented as min to max box and whisker plot. *p<0.05

**SUPPLEMENTAL FIGURE 6**

**

**

**Supplemental Figure 6. Additional behavioral parameters.** The behavior of control and Nav1.8-TSC2^KO^ mice at 8-weeks of age was tested in the elevated plus maze (EPM) and open field. **(a)** EPM, number of entries into the open and closed arms. **(b)** Total distance traveled during the full EPM evaluation including all available spaces. **(c)** Open field, distance traveled by location during the 60-minute period. **(d)** Open field, total distance traveled in 60-minutes. Males and females combined. 2-way ANOVA with Sidak’s multiple comparisons test. Sample sizes as indicated on the graphs. Presented as min to max box and whisker plot. *p<0.05
